# Water Dressing

**Published:** 1855-03

**Authors:** Geo. H. Hubbard


### WATER DRESSING.

BY GEO. H. HUBBARD, M. D.

The following cases are selected to illustrate the success attending the treatment of wounds with water. Although not of great importance as "interesting cases," if their publication can induce any one to abandon the burdensome and too often injurious appliances which have been customary in times gone by, and to substitute therefor treatment by water, a mode of treatment sanctioned by the best surgeons of the present day, perhaps as much good may be accomplished as by a lengthy detail of more intricate and unusual diseases.

In some notes on "water," which I furnished for a previous volume of this Journal, I entered somewhat into the history of its use as a therapeutical agent. At this time I purpose only to give a few cases in proof of the propositions then advanced, that "water is our best dressing in wounds of all kinds."

### CASE I.—*Incised Wound of the Knee.*

D. P,, aged 18, made a mis-step while mowing, and in falling struck his knee on the scythe, which rested on the ground with its edge so inclined that the whole weight of his body came upon it at right angles. It was consequently forced into the limb as far as such a blow could drive a sharp edge into bone. The resulting, clean, incised wound severed the ligamentum patellæ, about midway of its length; not directly across, but obliquely, its internal angle being just one inch lower down the limb than the external.

The wound was precisely four and seven eighths inches in length. The capsule of the joint was opened by a clean incision, seven-eighths of an inch long at the external angle of the wound, and a piece of the head of the tibia was split off and slightly movable at the inner angle; but as it could not be removed without cutting it from the ligaments of the joint to which it was attached, it was placed in as good coaption as possible, and allowed to remain.

When I reached the patient (about an hour after the injury,) he was considerably depressed, perhaps as much, or more, from the fright which this gaping wound was calculated to produce, as from the injury. As no important branches of the anastomosing arteries were cut, the hæmorrhage was slight, and that from the small cutaneous vessels had nearly ceased on my arrival. The lower portion of the ligamentum patellæ, which protruded prominently from the wound, was, after removing some bits of grass, brought into exact coaptation, and kept so by means of the twisted suture, consisting of three steel needles, deeply inserted, one and one-fourth inches apart. The limb was laid in a "Goodwin's splint," and kept perfectly extended.

The wound was dressed in *water*, and was never dry for twenty-five days. The temperature of the water was gradually raised after the expiration of ten days, and regulated by the sensations of the patient; keeping it just so cold as was agreeable. For the first week his diet was *water gruel*, afterwards, additions were made, very gradually, so that he did not receive full liberty to eat as he chose, for five weeks.

After this wound was first dressed, he had "*no pain*," lost no sleep, and there was but little swelling about the wound. No fever occurred; the wound united by first intention, except a small hole which kept open at the inner angle for about five weeks; doubtless on account of injury done the bone at that point; but at the end of this time it was entirely healed. Two of the needles were removed on the eighteenth day, and the third on the twenty-first. On the fortieth day, he walked two miles with the aid of a small stick.

No medicine of any sort was at any time administered; the water was discontinued on the twenty-fifth day, and an envelope of cotton used to protect the knee both from injury and from cold.

At this time, fourteen months after the injury, he is walking without the aid of a cane, engaged in all sorts of farm-work, having rendered good service on the farm during the past season.

CASE II.

P. P., aged 20, while chopping wood, misguided his axe, and received its full force on the dorsum of the left foot. Taking a somewhat slanting direction it did not go through the foot, as a perpendicular blow of like momentum wonld have done, but split up portions of the tarsal and metarsal bones; so that when I examined the foot, about an hour after, I introduced my fingers to the depth of an inch and a half, removing the clotted blood and some small fragments of bone which were entirely separated. The wound was strapped tightly with strips of linen wet with collodion, and a compress bound on to keep the split portion of the bone in as good coaptation as possible. A splint, to keep the foot up so as to relax the muscles acting upon its anterior aspect, was used, as many of the extensor tendons were severed. This wound was dressed with "*water, and nothing else;*" was never dry for two weeks; the temperature was raised as the sensations of the patient indicated; it united by first intention throughout its whole extent; there was no suppuration; the bones seemed to unite as readily as the integuments, and on the 28th day he went to his work with but slight lameness, from which he entirely recovered.

### Case III.

A girl, aged 10 years, tried her hand at chopping wood, and, from want of skill and strength, cut her foot severely. Upon examining the wound, I found the great toe nearly severed from the foot, hanging only by the skin, flexor tendons, and vessels of its inferior part. The metatarso-phalangeal articulation was laid entirely open, and the whole surface of the metatarsal bone cut off and loose in the wound, from which it was removed by snipping with scissors some few ligamentous fibres which had escaped the axe. The wound was cleaned, and all loose portions of bone and clots of blood removed. When this was done, the bleeding, which had been profuse, ceased entirely. This wound was brought together by interrupted sutures, and the toe brought into as good shape as possible, and kept so by bandages. Water was the only application made, and to the surprise of all, no pain was felt, no sleep lost; the wound united by first intention, and she has a very good and servicable toe. I am inclined to think excision of a joint was never more successful than in this case, nor the operation ever performed with a ruder instrument or less surgical skill.

### Case IV.

A man came to me, with a finger the knuckle joint of which he had brought in contact with a circular saw. I found a ragged wound, which had opened the joint and removed portions of cartilage from the articulating surfaces of the bones. After removing all loose substance, the wound was brought together and dressed with collodion and strips of linen. He was ordered to keep it constantly wet with water, of such temperature as was agreeable, with which direction he faithfully complied. I saw the finger occasionally; but as no inflammation or suppuration occurred, I had no more to do with the case, and the first dressings were not removed till the wound was cured. The finger was nearly as serviceable as ever, the joint being but slightly stiffened.—*New Hampshire Journal of Medicine.*

